# The Role of Genetic Testing in Palliative Care Decisions for Critically Ill Newborns

**DOI:** 10.3390/children12050634

**Published:** 2025-05-15

**Authors:** Ashley Mowery, Luca Brunelli

**Affiliations:** Pediatrics/Neonatology, Spencer Fox Eccles School of Medicine, University of Utah, Salt Lake City, UT 84112, USA; ashley.mowery@hsc.utah.edu

**Keywords:** genetics/genomics, palliative care, neonatal intensive care, newborn

## Abstract

Genetic testing is rapidly becoming standard practice in the care of critically ill newborns within NICUs. Numerous studies have demonstrated the utility of genetic testing, including changes in clinical care, improved diagnostic certainty, and cost savings, related to a reduced length of hospital stay. Changes in clinical management reported in previous studies also included redirection to comfort or end-of-life care. However, it has been difficult to study the influence of genetic testing in the redirection of care decisions within the NICU because of the complexity of the medical decision-making process. Redirection of care decisions are deeply personal for each individual family and often must be made in the setting of clinical instability and diagnostic and prognostic uncertainty. A recent study exploring the impact of genetic testing in redirection of care decisions by surveying palliative care providers suggested genetic testing plays a minor role in decisions to redirect to end-of-life care or in the implementation of DNR/DNI orders. However, factors such inadequate treatment options were found to be important in redirection of care decisions, implying the need for further investigation to clarify the role of genetic testing. Future studies will need to focus on how genetic information affects healthcare provider recommendations regarding palliative care and how families use this information to make end-of-life care decisions.

## 1. Genetic Testing in the NICU

Genetic and congenital anomalies affect approximately “6% of live births” and are a major cause of hospital admission and mortality [1]. Newborns with genetic conditions often experience longer hospital stays and greater resource utilization [1]. Morbidity and mortality associated with genetic diseases are more notable in level IV neonatal intensive care units (NICUs) because they care for the most at-risk and severely ill newborns [2]. In the last 15 years, genetic testing has transformed the ability to diagnose newborns in NICUs. A report that reviewed the clinical data of 256 infants admitted to a level IV NICU in anticipation of introducing rapid genetic testing found that “a fifth of infants admitted to a level IV NICU might be eligible” for rapid next-generation sequencing (rNGS) and “38% of eligible infants might be successfully diagnosed” using rNGS [2]. rNGS was defined as targeted gene panels, exome sequencing, or whole-genome sequencing that produce results within approximately one week [2].

Many studies have investigated the clinical utility of genetic testing in the care of critically ill newborns [3]. Studies have suggested genetic testing leads to changes in medical care, including new treatments, types of procedures performed, subspecialty care provided, and pursuit of palliative care or end-of-life care [1,4,5,6]. Others reported the benefits of genetic testing include improved diagnostic and prognostic certainty [4,7]. The information provided by rapid whole-genome sequencing (rWGS) could help inform acute and long-term management strategies, anticipate possible comorbidities, and guide pregnancy planning and counseling based on risk of recurrence [4,7].

A multicenter randomized trial conducted from 2017 to 2019 included 354 infants with a suspected genetic condition, 176 randomized to an “early” group who received their genetic test results 15 days after enrollment and 178 randomized to a “late” group who received their genetic test results 60 days after enrollment [5]. This study demonstrated that at least 20% of critically ill infants tested with rWGS had changes in clinical management, including referrals to subspecialists, invasive procedures, and alterations in medications [5]. Infants with pathogenic genetic test results were “more than 3-fold more likely to receive a [change of management] compared to those with uncertain or negative findings” [5]. However, this study did not identify a difference in length of hospitalization or mortality [5].

“Project Baby Bear” was a large multicenter study aimed at implementing rWGS within the ICUs of five children’s hospitals across California [6]. The study focused on changes in clinical management based on rWGS results and cost of care. They reported 32% of acutely ill newborns with rWGS had changes to their clinical care [6]. Similarly to previously published data, these changes included adjustments to infants’ procedures, medications, and nutrition [6]. The cost associated with rWGS in this study was USD 1.7 million, but the study estimated rWGS led to USD 2.2–2.9 million cost savings in regard to “hospital costs and professional fees” incurred when caring for critically ill infants [6]. Reportedly, 89–93% “of estimated savings derived from reduced length of stay” and the other 7–11% of savings were related to “avoided diagnostic tests” [6]. In several cases, reduced length of stay was due to infants undergoing redirection of the goals of care and withdrawal of life-sustaining measures [6].

One study focused on the use of rWGS in the NICU and families’ opinions of genetic testing found that “although only 23% of infants received a rWGS diagnosis, 97% of parents reported that genomic testing was at least somewhat helpful” [4]. Reportedly, 80% of parents felt “genomic sequencing made them more knowledgeable about their child’s future health” [8]. More specifically, the study reported that “90% who received a positive genomic diagnosis perceived the test to be beneficial to their child relative to those who received negative results” [8]. When comparing parental and provider perceptions of the utility of genetic testing, “only 4% of clinicians” reported genetic testing was useful when parents did not [8]. A mixed methods retrospective study further explored the perspectives of parents regarding their infants’ genetic testing [9]. They reported positive attitudes toward genetic testing related to “hope of a diagnostic answer” that would guide their child’s medical care [9]. They also described that parents with neutral or negative attitudes toward genetic testing were related to “fears about what the results would reveal about their child’s illness” [9]. Concerns have been raised that parents may pursue genetic testing in an attempt to have more certainty without considering the short- and long-term implications associated with genetic testing and its results [10]. This study addressed the concern that parents may not fully understand the risks associated with genetic testing. Reportedly, “the widespread availability of consumer-driven genetic testing may suggest that some risks” are not regarded “as serious by patients and families as they are by clinicians” [9]. Regardless, these studies suggest that the use of genetic test results in medical decision making, including palliative care or redirection of care decisions, must incorporate the values of the patient’s family and include shared decision making between the infants’ parents and clinicians [9,11].

## 2. Neonatologist Involvement in Genetic Testing Within NICUs

Neonatologists are in a unique position regarding genetic testing in critically ill newborns because they do not have formal training in genetics, like medical geneticists or genetic counselors. One study surveyed 551 neonatologists across the country to further understand how they counsel and utilize genetic testing. Surveys demonstrated that genetic testing is performed on “5–25% of patients”, and approximately 19% of providers do not have medical geneticists on site [11]. Across the country, there has been a shortage of medical geneticists and genetic counselors, which has led to neonatologists and NICUs finding innovative ways to utilize genetic testing. Surveys found that 81% of providers counsel and obtain consent for genetic testing in the NICU, and 95% of providers are responsible for discussing genetic test results despite some neonatologists feeling uncomfortable due to the lack of support from dedicated medical genetics teams [11]. Another study surveyed forty-three level IV NICUs to establish the availability of genetic testing and medical geneticists, in addition to barriers preventing the utilization of these resources. Surveys found that 69% of included level IV NICUs had access to whole-exome sequencing or whole-genome sequencing [12]. Regarding barriers to utilizing genetic testing, the survey reported 47% was related to the need for parental samples, 41% was due to the counseling and informed consent process, and 13% was due to the inability to access medical geneticists or genetic counselors [12]. While neonatologists often provide counseling prior to genetic testing, surveys found that 91% of level IV NICUs involved a medical geneticist or genetic counselor in the counseling that occurs after genetic test results are returned [12].

Genetic testing may provide valuable diagnostic and prognostic information, but it may also yield equivocal data [13]. Specifically, there may be hesitation regarding uncertain genetic test results, which “may be unrelated” to a newborn’s “phenotype or have unclear implications for treatment [13]. A randomized controlled psychological experiment focused on neonatologists’ clinical decision making regarding genetic testing surveyed 551 neonatologist using experimental clinical cases that involved genetic test results with “uncertain prognostic value” or were “associated with a range of clinical outcomes” [13]. For the clinical case with a variant of uncertain significance, neonatologists were “less likely to recommend tracheostomy and gastrostomy tube” and were “more likely to recommend transitioning to palliative care” [13]. Neonatologists also reported “pain and suffering” as a more important factor in clinical decision making; thus, they were “less likely to recommend invasive care” [13]. Notably, medical geneticists recommend variants of uncertain significance not be involved in clinical decision-making processes. Surveys also demonstrated that the identification of incidental genetic test abnormalities associated with neurodevelopmental outcomes led to palliative care interventions being favored over invasive care [13]. Another clinical case demonstrated that the diagnosis of genetic disease corresponded to “an increased likelihood of recommending palliative care” [13]. Lastly, these surveys reported a genetic diagnosis most influenced neonatologists’ recommendations to the newborn’s family. However, genetic information may be ambiguous and should be used with caution in medical decision making [13].

These studies further support the need to empower “neonatologists with basic genetic counseling skills” to ensure safe access to genetic testing for critically ill newborns [12]. Additional training in genetics may also address concerns regarding the consent process and interpretation of genetic test results. One study focused on providing limited genetics education to NICU providers conducted in-person training sessions involving presentations, group discussions, and simulations [14]. The training focused on the benefits and limitations of genetic testing, gaining familiarity with genetic test reports, and recommendations regarding how to discuss genetic test results with families. The training did not provide education regarding “comprehensive genetic counseling” or “interpreting secondary or incidental results” [14]. Of the 26 physicians and 7 nurse practitioners that participated in the study, more than 50% of providers did not have formal training in medical genetics. Following in-person training, providers reported increased confidence in “their ability to review, understand, and use genome sequencing results” in the diagnosis of genetic diseases and clinical decision making [14]. While this study demonstrates additional training of “non-genetics providers” may be beneficial, there are limitations to the large-scale implementation of standardized genetics training [14]. The development of this training would require significant time and effort by experts in medical genetics, and adapting the training into various forms of continuing medical education (CME) would be essential [14].

## 3. Genetic Testing and Palliative Care in the NICU

“Project Baby Bear” reported USD 2.2–2.9 million cost savings related to reduced length of hospital stay, in several cases resulting from infants undergoing redirection to comfort or end-of-life care [6]. The study assumed that redirection of care in infants tested with rWGS was due to genetic tests [6]. However, decisions to redirect care are often complex and, therefore, difficult to ascribe solely to one cause. Given the complexity of decision making in the NICU, the specific impact of genetic testing on decisions to pursue redirection to end-of-life care has been difficult to discern [3]. It is, therefore, important to better understand the role of genetic testing in these situations.

Relatively few studies have commented on the potential impact of genetic testing in palliative care decision-making processes, including redirection to end-of-life care. One study focused on the economic impact of rWGS in the pediatric ICU stated genetic “information empowered parents to shift their goals for clinical care to comfort rather than curative or prolonged care” [4]. Another study concentrating on the utility of rWGS in regard to improving patient outcomes reported “rWGS facilitates end-of-life care decisions” by reducing suffering and assisting in grieving [1]. Lastly, a study focused on the utility of exome sequencing reported “informed redirection of care (including palliative care and withdrawal of life support) was undertaken in 35.8%” of critically ill infants with “serious disorders” [15]. An analysis of the utility of genetic testing in infants reported that genetic diagnoses often precede decisions to pursue end-of-life care, which makes it “difficult to determine whether the diagnoses alone would have justified such decisions” or “whether the clinical course could have been predicted without [genome sequencing]” [16]. A literature review focused on parents’ engagement in end-of-life care decision-making processes for NICU infants reported that families’ decisions to redirect to end-of-life care are individualized and are often “based on future quality of life, severity of handicaps, and projections of longevity and often reflect the values of parents and healthcare providers” [17]. Given the complexity, uncertainty, and individualized nature of these decisions, the implications of genetic testing in palliative care decision-making processes have been unclear and largely understudied until recently [3,16,18].

## 4. A New Perspective on the Role of Genetic Testing in Palliative Care Decisions

Palliative care teams are integral to the care of critically ill newborns in the NICU for numerous reasons, including assisting families through difficult redirection of care decisions [3,19]. Most studies, thus far, have not included palliative care providers and instead focused on understanding the perspectives of neonatologists and families towards genetic testing utilization and its impact on medical decision making. However, a recent retrospective study that included 56 infants admitted to a level IV NICU aimed to describe the impact of genetic testing on redirection of care decisions by surveying palliative care providers involved in the infants’ care [3]. Palliative care providers were surveyed to provide a more longitudinal perspective of an infant’s redirection of care process since other providers involved in their care such as the neonatologist often engage in shift work. Therefore, multiple neonatologists may care for an infant within the same week, limiting their ability to comment on the entire redirection of care decision-making process since decisions often occur over multiple days to weeks [3].

Surveys demonstrated that in 90% of redirection of care discussions, the severity of the infant’s illness, the family’s values, and the family’s perspective of quality life were discussed. The opportunity to pursue genetic testing was discussed in approximately 50% of redirection of care conversations [3]. Highlighting the increasing utilization of genetic testing in the NICU, 73% of infants who underwent redirection of care in this study received genetic testing, including WGS, rWGS, WES, microarrays, targeted gene panels, and chromosomal analyses [3]. For infants who did not receive genetic testing, the most commonly reported reason was that genetic testing was not necessary per clinical guidelines. For infants with genetic testing, 44% had positive genetic test results, 32% had negative genetic test results, and 22% had uncertain genetic test results [3].

Palliative care providers identified the most important value and belief involved in families’ decisions to pursue end-of-life care was the severity of the infant’s illness and “their pain and suffering”, regardless of genetic test results (positive, negative, or uncertain) [3]. The least important value and belief involved in decisions to pursue redirection of care identified by palliative care providers was the patients’ families’ faith and spirituality [3]. Palliative care providers identified “lack of medically appropriate treatment options” described as lack of medical or surgical interventions to treat the infants disease or the availability of only symptomatic management as the most important clinical component in families’ decisions to pursue redirection to end-of-life care, regardless of genetic test results (positive, negative, or uncertain) [3]. The second most important clinical component in decisions to pursue redirection of care regardless of genetic test results was the infant’s neurodevelopmental prognosis. A poor neurodevelopmental prognosis was described as development of learning or intellectual disabilities, behavioral disorders, or cerebral palsy [3]. Both clinical factors were ultimately up to individual interpretation by each palliative care provider as they completed each infant’s survey. The least important clinical factor identified by palliative care providers for infants with negative or uncertain genetic test results was the information and prognostication based on genetic test results [3]. The least important clinical factor identified by palliative care providers for infants with positive genetic test results was the infants’ code status and desires for code interventions [3].

Palliative care providers were directly asked about the impact of genetic testing on redirection of care decisions, and survey results demonstrated a relatively minor effect, although the effect was more prominent for infants with positive genetic test results [3]. Similarly, when directly asked about the impact of genetic testing on do-not-resuscitate and/or do-not-intubate orders, the effect was minor but more prominent in those with positive genetic test results [3]. Factors such as “lack of medically appropriate treatment options” may be influenced by genetic test results and require future investigation to better understand the role of genetic testing in redirection of care decisions [3]. Further complicating matters, more than 50% of newborns who underwent genetic testing had either negative or inconclusive results [3]. Nevertheless, this recent study highlights the complexity in trying to understand how genetic information informs redirection of care decisions.

## 5. Does Genetic Testing Facilitate Redirection of Care Decisions and Cost Savings?

Some experts suggest that, as genetic testing becomes more common, “it is likely that physicians and families will be more and more unwilling to redirect care to palliation without a concrete and definite diagnosis, even in cases where clinical evaluation has led to the conclusion” the infant’s disease is incurable [16]. The increased reliance on genetic testing in palliative care decision-making processes could potentially lengthen the redirection of care process in settings that do not have genetic testing readily available [16].

On the other hand, the recent survey of palliative care providers challenges the commonly held belief that decisions to redirect care in newborns who received genetic testing, including rWGS, are directly and solely due to those genetic test results. Several studies have reported that reduced length of hospital stay led to cost savings and suggested that genetic testing provided parents with greater diagnostic and prognostic certainty, enabling them to choose earlier whether to pursue life-sustaining or palliative/end-of-life care. However, if genetic test results are not the main driver of redirection of care decisions, then these findings also challenge the assumption that genetic testing directly leads to significant cost savings. Therefore, further investigations into the impact of genetic testing on redirection of care decisions in the NICU are essential as rWGS increasingly becomes standard in the care of critically ill newborns and ethical concerns continue to arise.

## 6. The Future of Genetic Testing in Newborns

Genetic testing has been mostly utilized in situations where a newborn has a suspected genetic diagnosis or unusual disease presentation. However, over time, genetic testing has slowly become more widely available and less targeted [20]. With the expanded capabilities of genetic testing and improving cost associated with analysis and interpretation, some have suggested “genomic-based” newborn screening (NBS) should be developed [21]. Genetic-based newborn screening could greatly increase the number of genetic diseases diagnosed and treated in newborns [21,22,23]. A study focused on the feasibility and scalability of genetic based newborn screening reported that the expansion of genetic testing has created “large databases of variant pathogenicity assessments”, which could be useful in deciding what variants to include in newborn screening [22]. This study estimates that approximately “1000 genetic disorders may meet criteria for NBS by 2030” [22]. Improving the ability to diagnose genetic diseases could also promote the development of targeted treatments [21,22]. Genomic-based NBS reportedly could be more advantageous in the care of “premature and low birthweight newborns” considering the current biochemical newborn screening often results in “false positives and negatives” in these newborns [22]. Another multicenter retrospective study investigating “newborn screening with target sequencing (NESTS)” emphasized the usefulness of disclosing carrier status for various diseases [24]. It is estimated that each person has approximately “2.8 pathogenic variants in recessive disease genes”, and knowledge of carrier status could impact recommended follow-up and recurrence risk stratification, which may affect future reproductive decisions for parents and the newborn [24]. Some experts have suggested that, in addition to identifying genetic diseases, genomic-based NBS could also potentially help identify treatable congenital infections, therefore preventing significant morbidity [23]. For example, approximately 10–15% of newborns with congenital CMV who are asymptomatic already have hearing loss at birth [23].

Despite the promising potential impact of genomic-based NBS, it would require significant investment in infrastructure, education, and informed consent. One concern raised is whether samples should be retested if a “treatment becomes available for a previously untreatable disorder” [23]. This also leads to ethical concerns centered around the potential misuse of genetic data and creating undue psychological stress [23]. However, the NESTS study reported that 95% of parents experienced an improvement in their anxiety and psychological stress after receiving counseling regarding their newborns’ genetic test results [24]. Overall, future studies are needed to understand the impact of genetic testing in NBS, similarly to the need for future studies into the effect of genetic testing on clinical decision making in the care of critically ill infants.

## 7. Conclusions

Genetic testing plays an increasingly important role in providing rapid diagnosis and beneficial changes in the clinical management of critically ill newborns. These changes have included redirecting care to comfort or end-of-life care, which are particularly challenging and complex decisions in the NICU. Although several studies have explored the clinical utility of genetic testing, it has been difficult to analyze the specific influence of genetic testing in redirection of care decisions within the NICU. A recent study surprisingly suggests that the influence of genetic testing on redirection of care decisions may be less than previously assumed. Although positive genetic test results played a larger role in decisions to redirect to end-of-life care compared to newborns with either negative or inconclusive genetic test results, more than 50% of newborns undergoing genetic testing had either negative or inconclusive results [3]. Therefore, it will be important to perform future studies aimed at better defining the relationship between genetic testing, especially uncertain genetic variants, and redirection of care decisions in critically ill newborns. These studies should include infants receiving palliative care before potential decisions to pursue end-of-life care are made as well as prospective surveys of multiple healthcare professionals and families. These analyses would provide a more comprehensive understanding of how genetic information affects the recommendations of healthcare professionals in complex cases.

Future research aimed at clarifying the use of genetic test results in clinical decision-making processes is also important considering neonatologists are increasingly responsible for counseling and consenting for an infant’s genetic testing and discussing genetic test results due to the lack of access to medical geneticists or genetic counselors. One study involving theoretical clinical scenarios reported neonatologists are more likely to recommend palliative care over invasive interventions in the setting of genetic variants of uncertain significance, genetic variants associated with varying neurodevelopment outcomes and genetic diagnoses [13]. Considering neonatologists are often not formally trained in medical genetics, participating in basic genetics education may provide some benefit, including increasing providers’ competency in counseling, consenting and interpretation of genetic test results, thus reducing the effects ambiguous genetic data have on clinical decision-making processes [14].

Future studies are also needed to further understand what information families take into consideration when making difficult end-of-life care decisions [3]. A unique area that has yet to be explored is how prenatal genetic diagnoses and postnatal genetic diagnoses influence parents’ decisions to pursue redirection of care or invasive interventions, for example, understanding whether parents with newborns diagnosed with a genetic condition prenatally are more or less likely to pursue redirection to comfort or end-of-life care compared to infants diagnosed postnatally. While this is an intriguing area of research, it may be difficult to investigate this relationship due to the need to compare similar phenotypes in newborns diagnosed prenatally and postnatally. The information gathered from these studies may also address some of the ethical concerns and challenges associated with the future of genetic-based NBS, which has the potential to increase the number of treatable diseases screened for at birth and promote the continued development of targeted treatments [21,22,23].

## Abbreviations

NICU	neonatal intensive care unit
rNGS	rapid next-generation sequencing
rWGS	rapid whole-genome sequencing
WGS	whole-genome sequencing
WES	whole-exome sequencing
DNR	do not resuscitate
DNI	do not intubate
NBS	newborn screening
CME	continuing medical education
